# Metastatic Castration-Resistant Prostate Cancer, Immune Checkpoint Inhibitors, and Beyond

**DOI:** 10.3390/curroncol30040323

**Published:** 2023-04-19

**Authors:** Sree M. Lanka, Nicholas A. Zorko, Emmanuel S. Antonarakis, Pedro C. Barata

**Affiliations:** 1 Deming Department of Medicine, Tulane University, New Orleans, LA 70112, USA; slanka@tulane.edu; 2 Department of Hematology and Oncology, Masonic Cancer Center, University of Minnesota, Minneapolis, MN 55455, USA; 3 Department of Hematology and Oncology, University Hospitals Seidman Cancer Center, Case Western Reserve University, Cleveland, OH 44106, USA

**Keywords:** immune checkpoint inhibitors, metastatic castration-resistant prostate cancer, immunotherapy combinations

## Abstract

The therapeutic landscape of several genitourinary malignancies has been revolutionized by the development of immune checkpoint inhibitors (ICIs); however, the utility of immunotherapies in prostate cancer has been limited, partly due to the immunologically “cold” tumor terrain of prostate cancer. As of today, pembrolizumab is the only immune checkpoint inhibitor approved for the treatment of metastatic castration resistant prostate cancer (mCRPC) in a select group of patients with high microsatellite instability (MSI-H), deficient mismatch repair (dMMR), or high tumor mutational burden (TMB). Looking ahead, several combinatorial approaches with ICIs involving radioligands, radiotherapy, PARP inhibitors, interleukin inhibitors, and cancer vaccines are exploring a potential synergistic effect. Furthermore, B7-H3 is an alternative checkpoint that may hold promise in adding to the treatment landscape of mCRPC. This review aims to summarize previous monotherapy and combination therapy trials of ICIs as well as novel immunotherapy combination therapeutic strategies and treatment targets in mCRPC.

## 1. Background

As a major public health concern, prostate cancer is a leading cause of cancer worldwide and the second leading cause of cancer death in men in the United States. In 2022, prostate cancer had an incidence of 268,490 and a 5-year-relative survival of 96.8%. However, in patients with distant metastases, the 5-year-relative survival dramatically decreased to 32.3% [1]. Although the treatment strategies for prostate cancer have progressed significantly in recent decades, the expected survival for patients with metastatic castration-resistant prostate cancer (mCRPC) remains poor. Immunotherapies targeting programmed death-1 (PD-1)/programmed death- ligand 1 (PD-L1) and cytotoxic T-lymphocyte-associated antigen 4 (CTLA-4), otherwise known as immune checkpoint inhibitors (ICIs), have revolutionized the therapeutic landscape of genitourinary malignancies such as renal cell carcinoma and urothelial carcinoma. However, they have not yet been shown to be quite as efficacious in mCRPC [2].

As an immunologically “cold” malignancy, prostate cancer may have had limited benefits from ICIs in previous trials due to low tumor burden, low major histocompatibility class I expression, dysfunctional interferon signaling, and a complex tumor microenvironment [3,4]. However, there is an unmet need for the personalization of treatment in mCRPC and although ICI monotherapy is not as efficacious, there may be utility in exploring combination therapies with ICIs and other targeted treatments. The utilization of histologic-agnostic agents remains to be an area that is largely unexplored in prostate cancer, with the exception of pembrolizumab [5]. It is key to take into account the tumor landscape of mCRPC when developing new combination treatments or personalized treatment regimens for patients [6]. To combat the difficult terrain of mCRPC, novel combination treatment strategies and therapeutic targets are being evaluated to advance the use of ICIs in prostate cancer. The goal of this review is to summarize previous and ongoing trials of ICIs in mCRPC and discuss ICI combination strategies and new therapeutic targets for patients with mCRPC.

## 2. Selection of Trials

In this review, the authors aimed to review the existing literature on monotherapy and combination therapy trials of immune checkpoint inhibitors, specifically in the context of mCRPC. A literature search of existing trials was conducted by searching PubMed for all relevant publications from its inception to 5 April 2023. Titles and abstracts were screened for relevance and full texts of articles were analyzed for eligibility in the review. In addition, breakthrough results of more recent trials that were presented at conferences were also included. There was an emphasis on landmark phase II and III trials that elucidated the extent of the utility of ICIs in mCRPC. In addition, more recent studies that explored ICIs in combination with other agents in mCRPC were also included.

## 3. Monotherapy: Previous Trials

As of today, pembrolizumab, an ICI targeting PD-1, is the only Food and Drug Administration (FDA) approved ICI for prostate cancer, but it is only specifically approved for mCRPC patients with high microsatellite instability (MSI-H), deficient mismatch repair (dMMR, or tumor mutational burden (TMB) ≥10 mut/Mb. Furthermore, it is recommended only as a subsequent therapy for mCRPC patients who progressed on docetaxel and a novel hormonal therapy [7]. This was extrapolated from a phase II KEYNOTE-158 study that investigated the utility of pembrolizumab in patients with previously treated, advanced non-colorectal MSI-H and dMMR tumors [8]. This study observed objective response in 20 out of the 102 patients with a TMB ≥10 mut/Mb and 43 out of 688 patients with less than 10 mut/Mb. Pembrolizumab was approved for use in the treatment of patients with unresectable or metastatic MSI-H or dMMR solid tumors that had progressed on prior treatment in 2017 [7]. In addition, in a study of a cohort of 65 dMMR mCRPC patients, 19 were treated with anti-PD1 therapy and showed a PSA response rate of 65% and median PFS of 24 weeks [9].

There have been several trials that have investigated ICI monotherapies in mCRPC including pembrolizumab. However, studies have shown that there is a very limited response to single-agent checkpoint inhibitor therapy (Table 1). For instance, in KEYNOTE-199, 258 mCRPC patients who were previously treated with docetaxel and one or more targeted endocrine therapy were given pembrolizumab [10]. In this study, three cohorts of patients were evaluated with pembrolizumab monotherapy. This study also included two cohorts or patients who progressed on enzalutamide, who were given combination pembrolizumab and enzalutamide, an androgen receptor inhibitor. The pembrolizumab monotherapy group had low objective and PSA response rates; however, the patients who did respond to monotherapy tended to have more durable responses. Furthermore, in the enzalutamide and pembrolizumab groups, the overall response rate (ORR) seemed numerically better than the monotherapy group. However, the phase III KEYNOTE-641 trial, which evaluated pembrolizumab in combination with enzalutamide and ADT in mCRPC, was discontinued after an interim analysis showed no improvement in rPFS or OS [11]. In addition, ipilimumab monotherapy was studied in two large phase III trials in chemotherapy-naïve and in docetaxel-pretreated mCRPC patients; however, improved overall survival (OS) was not met in either study [12,13]. Ipilimumab did, however, prolong progression free survival (PFS) and PSA responses in a subset of mCRPC patients. There has also been a phase 1 study that investigated the use of atezolizumab in 35 mCRPC patients who had progressed on sipuleucel-T or enzalutamide, which showed a minimal PSA response rate of 8.6% [14]. Combination with enzalutamide was studied in the IMbassador250 trial [15]. Furthermore, nivolumab was studied in a phase 1 trial, which included patients with melanoma, non-small-cell lung cancer, mCRPC, renal cell carcinoma, and colorectal carcinoma [16]. In the prostate cancer subset of patients, however, there were no objective responses to nivolumab monotherapy.

## 4. Combination Strategies

There have also been trials that have investigated immunotherapy combination treatments in prostate cancer (Table 2). For instance, in the phase III IMbassador250 trial, atezolizumab plus enzalutamide compared to enzalutamide therapy alone was studied in 759 mCRPC patients. These patients had already progressed on abiraterone and were ineligible for taxane-based therapy [15]. This study, however, did not meet the primary endpoint of improved overall survival in unselected patients. Similar results were found in the phase III KEYNOTE-641 trial, which evaluated pembrolizumab in combination with enzalutamide and ADT in mCRPC, which was discontinued after an interim analysis showed no improvement in rPFS or OS [11]. In another study, the phase II CheckMate 9KD trial, the utility of nivolumab and docetaxel was studied in 41 chemo-naïve mCRPC patients on ongoing androgen deprivation therapy (ADT) [17]. The ORR in patients with measurable disease was 36.8%, whereas the PSA response was 46.3%. It is thought that treatment with immunotherapy may augment the effects of docetaxel. There is now an ongoing phase III clinical trial, CheckMate 7DX to further investigate these results [18]. In cohort B of the KEYNOTE-365 trial, combination therapy with pembrolizumab plus docetaxel and prednisone was studied in chemotherapy-naïve mCRPC patients [19]. Out of the 104 patients who were treated, there was a PSA response rate of 28% with an ORR of 18%. The phase III KEYNOTE-921 trial evaluated pembrolizumab and docetaxel and prednisone in chemotherapy-naïve mCRPC patients [20]. The results of the KEYNOTE-921 trial were presented at the GU ASCO 2023 meeting, showing that the primary endpoints of rPFS and OS were not met [21]. There have also been trials to investigate dual immune-checkpoint inhibitor therapy including the phase II CheckMate 650 trial, which investigated the utility of ipilimumab and nivolumab in mCRPC patients who were previously treated with docetaxel [22]. It was shown that particularly in patients with high tumor mutational burden (TMB), ipilimumab and nivolumab combination therapy had clinical activity; however, treatment discontinuation occurred due to early toxicity. Additional results presented at ASCO GU 2023 included newly enrolled patients with an alternative ipilimumab and nivolumab regimen versus ipilimumab alone versus cabazitaxel. Several patients in the ipilimumab and nivolumab combination cohorts had a reduction (75–100%) in tumor size and PSA [23].

## 5. Novel Immunotherapeutic Targets beyond ICI

It is important to investigate whether the combination of immunotherapy with various other treatments could achieve synergistic effects in patients with prostate cancer (Table 3). To do this, it is essential to take a closer look at the complex tumor microenvironment in prostate cancer and identify other therapeutic targets that can be used alongside ICIs [24]. For instance, cytokines, specifically interleukins (ILs) such as IL-6, IL-8, IL-15, and IL-23, play a major role in the proliferation of cancer. Specifically in prostate cancer, IL-6 has been shown to be involved in radiotherapy resistance [25]. Although there have been several pre-clinical studies on therapies targeting cytokines in prostate cancer, there have not been many successful studies that have led to clinical outcomes [26]. In a phase II trial, siltuximab, a monoclonal antibody against IL-6, was evaluated in mCRPC patients who were previously treated with chemotherapy; however, there was only a 3.8% PSA response rate, and the results were largely disappointing [27]. There have not been many dedicated trials that have investigated combination regimens with interleukin inhibitors.

Furthermore, cancer vaccines such as sipuleucel-T were the initial backbone of prostate cancer management. There have been studies that have investigated the utility of sipuleucel-T with ICIs, for instance, the phase Ib study that studied a combination of sipuleucel-T with atezolizumab in 37 asymptomatic or minimally symptomatic mCRPC patients [28]. Of the 23 patients with measurable disease, only 4.3% had an objective response. Although the combination was well-tolerated, further studies are needed in larger cohorts to determine whether the combination is truly beneficial.

In addition, poly-ADP ribose polymerase (PARP) inhibitors, which work by interfering with DNA damage repair mechanisms, could be combined with ICIs. Even though there are studies that have investigated PARP inhibitor treatment such as olaparib and rucaparib, few have investigated their synergistic effects with ICIs [29]. However, cohort A of the KEYNOTE 365 study investigated pembrolizumab plus olaparib in 102 docetaxel-pretreated mCRPC patients with disease progression [30]. A total of 59 patients had measurable disease, with an ORR of 8.5%. Radiographic progression free survival (rPFS) was 4.5 months and the median OS was 14 months. Additionally, there is the KEYLYNK-010 phase III study, in which the pembrolizumab plus olaparib combination is being compared to enzalutamide monotherapy or abiraterone monotherapy in enzalutamide or abiraterone pretreated mCRPC patients who progressed on chemotherapy [31]. The primary endpoints being measured are overall survival and rPFS. The results of KEYLYNK-010 were presented at the European Society for Medical Oncology Congress in 2022, and it was shown that the primary endpoints of rPFS and OS were not met [32]. Furthermore, the phase II trial that investigated durvalumab, a PD-L1 inhibitor, and olaparib in mCRPC, showed a PSA response of more than 50% in 47% of patients [33]. This was especially notable in patients with DNA damage repair mutations, alluding that patients with these mutations may benefit further with a combination of immunotherapy and PARP inhibitor treatment.

Furthermore, although there have been a few studies looking into ICIs and radioligand therapy combinations, it may be worthwhile exploring this option as radioligand therapies could sensitize immunologically “cold” prostate tumors to ICIs [34]. The phase 1b/II PRINCE trial of pembrolizumab in combination with 177-Lu-PSMA-617 in mCRPC patients indicated a PSA response rate of 76% and an ORR of 78%. In addition, there is an ongoing phase II EVOLUTION trial introduced at ASCO GU 2023 that is investigating ipilimumab and nivolumab in combination with 177-Lu-PSMA-617. The primary endpoint is 12-month PSA progression-free survival. There are ongoing translational studies looking into the tumor microenvironment effects and predictors of response to this combination therapy [35]. There are further preclinical studies investigating the utility of actinium in addition to ICIs that are currently underway [36]. For instance, in the murine model study by Czernin et al., mice were treated with ^225^Ac-PSMA617, an anti-PD-1 antibody, or both. It was shown that combination therapy improved the time to progression and survival compared to monotherapy alone [36]. Finally, there have been many preclinical and phase II studies that have shown that radiotherapy in combination with ICIs can result in tumor regression. However, in subsequent phase III trials, there was no significant difference between the ICI plus radiotherapy. In a study that evaluated atezolizumab and radium-223 in mCRPC patients with bone and lymph node and/or visceral metastases, there was no clear clinical benefit of combination therapy [37]. In addition, the combination regimen had greater toxicity compared to either drug alone.

## 6. B7-H3 as an Alternative Immune Checkpoint in Prostate Cancer

In addition to exploring combination strategies with classical immune checkpoints (PD-1, PD-L1, CTLA4), an alternative approach is to validate additional checkpoints as potential therapeutic targets in prostate cancer. To this end, the B7 superfamily molecule B7-H3 (also known as PD-L3, or CD276) has emerged as a new target in prostate cancer [38]. Relative to PD-L1 and PD-L2, B7-H3 is expressed at much higher levels in prostate cancer [39] and is found in >80% of primary and castration-resistant tumor specimens [40]. While B7-H3 was initially proposed to be immunostimulatory, accumulated evidence supports its negative regulatory role for immune response [41,42]. Consistent with these findings, high B7-H3 expression is associated with lower CD3+ T cell density and higher Treg density and is inversely correlated with Black race (expression is higher in Caucasian patients relative to African ancestry patients) [43]. Interestingly, B7-H3 expression is strongly correlated with AR signaling and AR cofactors (FOXA1, HOXB13), and mechanistic studies have suggested that the B7-H3 promoter and distal enhancer regions are directly bound by AR and its co-factors, implying that B7-H3 expression is under AR transcriptional control [44]. In a recent neoadjuvant study using the B7-H3 targeting monoclonal antibody, enoblituzumab, in 32 patients with high-risk localized prostate cancer, B7-H3 inhibition produced several PSA reductions and Gleason score reductions while inducing the upregulation of CD8+ T cells, adaptive increases in PD-1/PD-L1, and the evidence of immune activation (elevated granzyme B, IFN-gamma signaling, and myeloid inflammation) [45]. Larger randomized adjuvant studies of enoblituzumab in high-risk prostate cancer are currently being designed. Finally, given its high expression across prostate cancer treatment states, B7-H3 is also being pursued by several groups as an antigen target without the goal of interrupting its immunoregulatory roles. Multiple strategies including antibody-drug conjugates (DS-7300 and MGC018), Tri-Specific NK cell engagers (TriKE), and chimeric antigen receptor CAR-T and CAR-NK cells are in various states of pre-clinical development and early phase clinical trials [46,47,48,49,50]. While not functioning via the typical mechanism for immune checkpoint inhibition by focusing on blocking the inhibitory role of immune checkpoint molecules, these strategies may provide a novel alternative for exploiting the high levels of B7-H3 expression in prostate cancer.

## 7. Discussion

Although monotherapy ICI trials in mCRPC have not been very promising, there are many trials that have combined ICIs with standard chemotherapy as well as targeted therapies. There is an unmet need for the personalization of treatment in mCRPC, and ICI combination therapies may play a role in targeted therapy for mCRPC patients. Except for pembrolizumab, tumor-agnostic drugs have not otherwise been approved in prostate cancer [6]. Utilizing other targets in the signaling pathway of the prostate cancer microenvironment can result in a synergistic effect with ICIs. For instance, as discussed, combining ICIs with chemotherapy, radioligands, radiotherapy, PARP inhibitors, interleukin inhibitors, and cancer vaccines holds promise in the future of mCRPC treatment. Not only that, but exploring different checkpoint targets other than the conventional PD-1, PD-L1, and CTLA-4 can create a novel treatment strategy that may benefit patients. Further large-cohort studies must be conducted on various combinations of ICIs to determine which patients benefit the most from different treatment targets.

## 8. Conclusions

In conclusion, even though immune checkpoint inhibitors have revolutionized the treatment landscape of many malignancies, they tended to have a lag in utility in mCRPC. In the setting of treatment resistance and immunologically “cold” characteristics of prostate cancer, it is important to delve further into the tumor microenvironment and identify additional targets for therapy and for combination regimens that sensitize tumors to ICIs. There have been several trials that have studied the efficacy and utility of ICIs in prostate cancer; however, few clinical trials have approved combination therapies. Certain combinatorial approaches with ICIs involving radioligands, radiotherapy, PARP inhibitors, interleukin inhibitors, and cancer vaccines could create a synergistic effect and may be the future of mCRPC therapy. In addition, alternative checkpoints (such as B7-H3) may hold more promise as better therapeutic targets in this disease.

## Figures and Tables

**Table 1:** Immune checkpoint inhibitor monotherapy evidence in mCRPC.

Trial Name/NCT	Phase	Patient Population	Treatment	Primary Endpoint	Results
CA184-095 (NCT01057810)	III	Chemotherapy-naïve patients with mCRPC without visceral metastases	Ipilimumab monotherapy	OS	28.7 months (95% CI, 24.5 to 32.5 months) in the ipilimumab arm versus 29.7 months (95% CI, 26.1 to 34.2 months) in the placebo arm (hazard ratio, 1.11; 95.87% CI, 0.88 to 1.39; *p* = 0.3667)
KEYNOTE-199 (NCT02787005)	II	Pretreated mCRPC patients in 3 cohorts (cohort 1 PD-L1 + disease, cohort 2 PD-L1 negative disease, cohort 3 bone predominant disease regardless of PD-L1 expression)	Pembrolizumab monotherapy	ORR	5% (95% CI, 2% to 11%) in cohort 1 and 3% (95% CI, <1% to 11%) in cohort 2
CA184-043 (NCT00861614)	III	Docetaxel-pretreated mCRPC patients	Ipilimumab vs. placebo after radiotherapy	OS	11.2 months (95% CI 9.5–12.7) for ipilimumab and 10.0 months (95% CI 8.3–11.0) for placebo (HR 0.85, 95% CI 0.71–1.00; *p* = 0.053)
CA184-095 (NCT01057810)	III	Asymptomatic or minimally symptomatic chemo-naïve patients with mCRPC	Ipilimumab vs. placebo	OS	28.7 months (95% CI, 24.5 to 32.5) for ipilimumab and 29.7 (95% CI, 26.1 to 34.2) in placebo (HR 1.11; 95% CI 0.88 to 1.39; *p* = 0.3667)
PCD4989g (NCT01375842)	Ib	mCRPC patients who have progressed on sipuleucel-T or enzalutamide	Atezolizumab monotherapy	Safety and tolerability of atezolizumab	Treatment- related adverse events in 60% of patients

Abbreviations: CI confidence interval; HR hazard ratio; mCRPC metastatic castration-resistant prostate cancer; ORR objective response rate; OS overall survival; PD-L1 programmed death-1 ligand 1.

**Table 2:** Immune checkpoint inhibitor combination therapy evidence in mCRPC.

Trail Name/NCT	Phase	Patient Population	Treatment	Primary Endpoint	Results
IMbassador250 (NCT03016312)	III	mCRPC patients who had progressed on abiraterone	Atezolizumab + enzalutamide vs. enzalutamide alone	OS	Stopped early due to low probability of trial achieving primary endpoint given risk of immune-mediated adverse events
KEYNOTE-641 (NCT03834493)	III	Chemo-naïve mCRPC patients who are abiraterone-naïve or are intolerant to or progressed on abiraterone	Pembrolizumab + enzalutamide vs. placebo + enzalutamide	OS, rPFS	Discontinued after an interim analysis showed no improvement in rPFS or OS
CheckMate 9KD (NCT03338790)	II	Chemo-naïve mCRPC patients with ongoing ADT and ≤2 prior novel hormonal therapies	Nivolumab and docetaxel with prednisone and then nivolumab	ORR, PSA response rate	Confirmed ORR (95% CI) was 40.0% (25.7–55.7), and the confirmed PSA_50_-RR (95% CI) was 46.9% (35.7–58.3)
CheckMate 7DX (NCT04100018)	III	Chemo-naïve mCRPC patients with ongoing ADT and ≤2 prior novel hormonal therapies	Nivolumab + docetaxel vs. placebo + docetaxel	rPFS, OS	Pending
KEYNOTE-365 (NCT02861573) Cohort B	1b/II	Chemo-naïve mCRPC patients who progressed on 4 weeks or more of abiraterone or enzalutamide	Pembrolizumab + docetaxel + prednisone	Safety, PSA response rate, ORR	Confirmed PSA response rate was 34% and the confirmed ORR was 23%. TRAEs occurred in 100 patients (96%). Grade 3–5 TRAEs occurred in 46 patients (44%). Seven AE-related deaths (6.7%) occurred (2 due to treatment-related pneumonitis)
KEYNOTE-921 (NCT03834506)	III	Chemo-naïve mCRPC patients who progressed on 4 weeks or more of abiraterone or enzalutamide	Pembrolizumab + docetaxel vs. docetaxel alone	OS, rPFS	Results presented at ASCO GU 2023 Conference: rPFS (median 8.6 mo with pembrolizumab + docetaxel vs. 8.3 mo with placebo + docetaxel; HR 0.85, 95% CI 0.7121.01; *p* = 0.0335) and OS (median 19.6 months vs. 19.0 months; HR 0.92, 95% CI 0.7821.09; *p* = 0.1677) were not met
CheckMate 650 (NCT02985957)	II	Asymptomatic/minimally symptomatic patients who progressed after 2nd-generation hormone therapy and have not received chemotherapy for mCRPC (cohort 1) and patients who progressed after taxane-based chemotherapy (cohort 2)	Ipilimumab + nivolumab	ORR; rPFS	Median rPFS (95% CI) in all treated patients was 5.5 (3.5–7.1) and 3.8 months (2.1–5.1) in cohorts 1 and 2. In patients with TMB above vs. below the median, the ORR was 50.0% (95% CI 26.0–74.0) vs. 5.3% (95% CI 0.1–26.0)
CheckMate 650 (NCT02985957) additional results	II	mCRPC patients previously treated with docetaxel	Nivolumab + ipilimumab q3weeks for 4 doses then nivolumab q4weeks (cohort 1) vs. nivolumab q3weeks for 8 doses and ipilimumab q6w for 4 doses then nivolumab q4weeks (cohort 2) vs. ipilimumab alone (cohort 3) vs. cabaziaxel (cohort 4)	ORR, PSA response rate, rPFS	ORR 9% (cohort 1) vs. 15% (cohort 2) vs. 4% (cohort 3) vs. 11% (cohort 4). PSA response rate 14% (cohort 1) vs. 18% (cohort 2) vs. 5% (cohort 3) vs. 24% (cohort 4)

Abbreviations: ADT androgen deprivation therapy; CI confidence interval; HR hazard ratio; mCRPC metastatic castration-resistant prostate cancer; ORR objective response rate; OS overall survival; PD-L1 programmed death-1 ligand 1; PSA prostate-specific antigen; rPFS radiographic progression-free survival; RR response rate; TMB tumor mutational burden; TRAEs treatment-related adverse events.

**Table 3:** Combination strategies and targets with ICIs in mCRPC.

Trial/NCT	Phase	Patient Population	Treatment	Primary Endpoint	Results
ICIs + cytokines					
SWOG S0354 (NCT00433446)	II	mCRPC patients with prior taxane therapy	Siltuximab every 2 weeks for 12 cycles	PSA RR defined as 50% reduction	Overall PSA RR of 3.8% (95% CI: 0.5%, 13.0%)
ICIs + cancer vaccines					
NCT03024216	1b	Asymptomatic or minimally symptomatic mCRPC patients	Atezolizumab followed by sipuleucel-T (Arm 1) or sipuleucel-T followed by atezolizumab (Arm 2)	Safety	At least one treatment-related AE was reported in 31 subjects (83.8%), including 7 (18.9%) with at least one grade 3 treatment-related AE
ICIs + PARP inhibitors					
KEYNOTE-365 (NCT02861573) Cohort A	1b/II	Docetaxel-pretreated mCRPC patients who progressed within 6 months of screening and were molecularly unselected	Pembrolizumab + olaparib	Safety, PSA response rate, ORR	The confirmed PSA response rates in patients with a baseline PSA measurement were 15% (15/102) for the total population and 19% (11/59) for patients with RECIST-ORR was 8.5% (five PRs) in patients with RECIST-measurable disease. All 102 treated patients (100%) experienced at least one all-cause AE, and grade 3–5 AEs occurred in 74 patients (73%) Treatment-related AEs occurred in 93 patients (91%)
KEYLYNK-010 (NCT03834519)	III	mCRPC patients who progressed after chemotherapy and either abiraterone or enzalutamide	Pembrolizumab + olaparib vs. next-generation hormonal agent	OS, rPFS	rPFS (median 4.4 months with pembrolizumab + olaparib vs. 4.2 months with next-generation hormonal agent; HR 1.02, 95% CI 0.82–1.25; *p* = 0.55) and OS (15.8 mo vs. 14.6 mo; HR 0.94, 95% CI 0.77–1.14; *p* = 0.26) were not met. Study was stopped for futility.
NCT02484404	II	mCRPC patients who had received prior enzalutamide and/or abiraterone unselected for somatic or germline mutations	Durvalumab + olaparib	rPFS, PSA response	9 of 17 patients (53%) had a PSA decline of ≥50%. Median rPFS for all patients is 16.1 months (95% CI: 4.5–16.1 months) with a 12-month rPFS of 51.5% (95% CI: 25.7–72.3%)
ICIs + radioligand therapies					
PRINCE (NCT03658447)	1b	mCRPC patients with high PSMA expression (SUVmax ≥20 in an index lesion, SUVmax >10 for all lesions ≥10 mm), and no FDG positive/PSMA negative lesions on paired baseline PET/CT	^177^Lu-PSMA-617 + pembrolizumab	Safety, PSA response rate	PSA response rate was 76% (28/37 [95% CI 59–88]) and 7/10 (70%) patients with RECIST-measurable disease had a partial response
NCT02814669	1b	mCRPC patients with bone and lymph node and/or visceral metastases that progressed after androgen pathway inhibitor treatment	atezolizumab + radium-223	Safety, ORR	All 44 patients had ≥1 all-cause AE; 23 (52.3%) had a grade 3/4 AE. 15 (34.1%) grade 3/4 and 3 (6.8%) grade 5 AEs were related to atezolizumab; none were related to radium-223. Confirmed ORR was 6.8% [95% CI, 1.4–18.7]
NCT05150236	II	mCRPC patients with progression on prior androgen receptor pathway inhibitors, no more than one line of prior chemotherapy, significant PSMA avidity on 68GaPSMA-11 PET/CT (SUVmax ≥15 at one disease site and SUVmax ≥10 at measurable sites of disease. 10 mm), no FDG positive/PSMA negative disease and no contraindications to ICI	Ipilimumab + Nivolumab + ^177^Lu-PSMA-617	12-month PSA PFS	Ongoing

Abbreviations: ADT androgen deprivation therapy; AEs adverse events; CI confidence interval; FDG [18] F-fluorodeoxyglucose; HR hazard ratio; ICIs immune checkpoint inhibitors; mCRPC metastatic castration-resistant prostate cancer; ORR objective response rate; OS overall survival; PD-L1 programmed death-1 ligand 1; PFS progression free survival; PR partial response; PSA prostate-specific antigen; PSMA prostate-specific membrane antigen; rPFS radiographic progression-free survival; RR response rate; SUVmax maximum standardized uptake value; TMB tumor mutational burden.

## Data Availability

Not applicable.
